# Donor-specific graft injury in solid organ transplantation: potential mechanisms and therapeutic strategies

**DOI:** 10.3389/frtra.2024.1427106

**Published:** 2024-06-19

**Authors:** Chengliang Yang, Casey P. Shannon, Hedi Zhao, Scott J. Tebbutt

**Affiliations:** ^1^ Prevention of Organ Failure (PROOF) Centre of Excellence, St Paul’s Hospital, Vancouver, BC, Canada; ^2^ Centre for Heart Lung Innovation, Providence Research, St Paul’s Hospital, Vancouver, BC, Canada; ^3^ Division of Respiratory Medicine, Department of Medicine, University of British Columbia, Vancouver, BC, Canada; ^4^ Department of Surgery, University of British Columbia, Vancouver, BC, Canada

**Keywords:** donation after brain death, donation after circulatory death, donor evaluation, primary graft dysfunction, inflammatory, cell death

## 1 Background

Solid organ transplantation saves the lives of patients affected by end-stage organ failure and improves their quality of life. Donor organ supply is limiting, however. Donation after circulatory death (DCD) donors are one of several donor pools utilized to overcome the problem posed by the shortage of donation after brain death (DBD) donors. DCD have increased dramatically over the past 20 years in the United Kingdom, from 42 donors in 2001–2002 to 612 donors in 2021–2022 (1). Similarly in the United States, DCD organ transplantation has increased from approximately 4,189 cases in 2021 to 4,776 cases in 2022 (2). Here, we review the current state of the literature on DCD vs. DBD organ transplantation and propose future avenues for research.

### 1.1 Heart transplantation

Due to the inevitable experience of warm ischemic injury before DCD organ retrieval, concerns about the presence of susceptibility to warm ischemic injury in the donor hearts are greater than in other organs. While very carefully selected DCD organs can have similar early outcomes to those of DBD organs (3), this had not been demonstrated in randomized controlled trials of heart transplantation. Recently, Schroder and colleagues reported in the *New England Journal of Medicine* that 6-month post-transplant survival of heart DCD using an extracorporeal perfusion system was non-inferior to standard DBD using traditional cold storage (4). As in a previous study from the United States (5), the incidence of primary graft dysfunction (PGD, a severe form of ischemia-reperfusion acute allograft injury) was significantly higher in DCD compared to DBD heart transplant recipients (22% vs. 10%) (4). Similarly, another recent study of heart transplantation from the United Kingdom showed a higher rate of PGD requiring postoperative extracorporeal membrane oxygenation (ECMO) after DCD compared to DBD (6). Earlier this year, Ayer and colleagues reported more frequent severe biventricular PGD in DCD vs. DBD heart transplant recipients, but surprisingly, noted that DCD recipients with severe PGD experienced fewer days in hospital and on mechanical circulatory support compared with DBD recipients (5). Emerging evidence suggests that DCD recipients are more likely to experience acute rejection and hospitalization for rejection after heart transplantation compared to DBD recipients (7). Metabolomic biomarkers in normothermic ex-vivo heart perfusion perfusate, such as long chain acylcarnitines (including C16, C18:1, C18:2, and C20:4), differed significantly between DCD hearts and DBD hearts, whereas long chain acylcarnitines were associated with lactate and cardiac troponin I, suggesting graft myocardial injury may be related to donor type (8). Despite these differences, recently published data show survival outcomes of DCD and DBD heart transplant recipients are similar (3–5).

### 1.2 Kidney transplantation

Similarly, in kidney transplantation, a systematic review and meta-analysis of 51 cohort studies with 73,454 DCD recipients and 518,229 DBD recipients has shown a higher risk of primary non-function, delayed graft function, and a 13% increased risk of graft loss in the first year after DCD kidney transplantation (9). Ten-year DCD kidney transplant outcomes, however, are similar to DBD (9). Moreover, a UK Transplant Registry cohort study of 6,490 kidney transplant recipients showed that prolonged cold storage reduced the survival of DCD kidney grafts. However, the duration of cold storage had no effect on the survival of DBD kidney grafts (10). In response to the drawbacks of static cold storage, a recent randomized controlled trial of DCD kidney transplantation has demonstrated that normothermic mechanical perfusion of circulating warmed, oxygenated red-cell-based perfusate through the kidney to maintain near-physiological conditions is feasible, safe and suitable for clinical application (11).

### 1.3 Liver transplantation

Graft loss and recipient mortality were about twice as high with DCD vs. DBD livers in the United Kingdom (12). Research teams in the United States and the United Kingdom found that DCD livers have a higher rate of primary non-function and a much higher rate of biliary complications (13, 14). However, researchers in the United States believe that carefully selected, underutilized DCD livers recovered from younger donors (age <50 years old) with short cold ischemia time have better outcomes than those from older donors (>60 years old) (15). A meta-analysis of 25 cohort studies (2,478 DCD recipients and 59,706 DBD recipients) conducted by UK researchers in 2014 reported that DCD liver transplantation was associated with increased biliary complications, ischaemic cholangiopathy, graft loss and mortality (16). Additionally, a United Network for Organ Sharing (UNOS) database analysis and publication bias-adjusted meta-analysis of 13 cohort studies of 5,620 DCD and 87,561 DBD liver transplant recipients conducted by researchers in the United States in 2022 showed no difference between DCD and DBD in terms of patient survival, while DCD was associated with an increased risk of graft loss (17). Interestingly, a comparative study from the United Kingdom showed that DCD livers are more prone to necrosis than inflammation compared to DBD allografts (18). The opportunity to use normothermic machine perfusion to assess isolated organ viability is particularly attractive for evaluating DCD livers or livers from high-risk donors (19). A randomized trial of normothermic preservation in liver transplantation showed that the protective effect of normothermic machine perfusion was significantly different between DCD donor livers and DBD donor livers, particularly concerning the peak level of serum aspartate transaminase (AST) within seven days post-transplant (20). AST, a clinically accepted biomarker, proved predictive of primary non-function, as well as graft and liver transplant patient survival (20). However, the predictive capability of measuring specific bile parameters during NMP assessment for non-anastomotic biliary strictures, a significant cause of morbidity, graft loss, and mortality post-liver transplantation, remains uncertain. In a recent study conducted in the United States, among donor livers producing bile during NMP, all DBD livers were successfully rescued, while only 50% of the bile-producing DCD livers achieved the same outcome (21). In liver transplant patients, elevated microRNAs (miRNAs) and the oxygen-sensing prolyl hydroxylase domain 1 (PHD1) repression were seen in post-ischemic biopsies. MicroRNA miR122 regulates gene expression and is key in liver IR injury. Overexpressing miR122 pharmacologically reduced liver injury. Targeting miR122 may reduce hepatic injury during transplantation. miRNAs could be targeted to enhance hepatic ischemia tolerance (22). These findings suggest that donor-specific graft injury may have implications for the overall function of the allograft. An urgent need exists for a comprehensive investigation to delve into the differences in parameters such as mean peak AST, bile production, and post-liver transplantation outcomes between DCD and DBD livers during *ex vivo* organ perfusion, coupled with an examination of their underlying mechanisms.

### 1.4 Pancreas transplantation

A meta-analysis of 4 studies, including 152 DCD pancreas recipients and 1,682 DBD pancreas recipients, showed that there was no significant difference in allograft survival between DCD and DBD pancreases (23). However, DCD transplants have a higher rate of thrombosis than DBD transplants (23). This may be related to warm ischemic injury, and *in vitro* evidence of islet cell injury suggests that hypoxia leads to central necrosis with apoptotic features such as nuclear pyknosis and DNA fragmentation (24). Presently, *ex vivo* organ perfusion technology enables the monitoring of organs and assessment of their quality before transplantation. This stands in contrast to clinical pancreas transplantation, where only a limited number of experimental and preclinical studies have been published (25). A research group from UK found that insulin secretion occurred in all DBD pancreases during *ex vivo* normothermic perfusion; however, the lowest levels were observed in DCD pancreases (26). Furthermore, they noted that pancreases from DCD donors displayed the lowest basal and glucose-stimulated insulin levels, with mean insulin levels significantly below those observed in DBD pancreases. Similarly, a Canadian research group observed an increase in insulin levels in the perfusate of only DBD pancreases during *ex vivo* normothermic perfusion in experimental settings (27). Importantly, both studies had limited sample sizes (25, 26), underscoring the need for future investigations with larger cohorts to thoroughly explore variations in outcomes between DCD and DBD pancreases.

### 1.5 Lung transplantation

The International Society of Heart and Lung Transplantation DCD Registry reported no significant difference in 5-year survival between DCD and DBD lung transplantation (28), and better 10-year survival outcomes for DCD lung recipients (29). A large retrospective study of 21,356 lung transplant recipients from the U.S. National Registry similarly showed no difference in survival time between DCD and DBD recipients (30). Interestingly, this study reported a higher rate of severe PGD (PGD3 at 72 h post-transplant) in DCD compared to DBD recipients, and 28% shorter median survival time in DCD with normothermic *ex vivo* lung perfusion (EVLP) compared to DBD without EVLP, possibly a result of significantly more smoking history, longer donor to recipient distance, and longer total cross clamp time in DCD with EVLP group (30).

## 2 Hypothesis of donor-specific injury

Alternatively, the observed differences could reflect cellular and molecular differences between EVLP DBD lungs and non-EVLP DBD lungs, including expression of the TNF family member receptors, TNF receptor (TNFR)-1/2 signalling pathways and macrophage migration inhibitory factor (MIF)-related pathways that support the inflammatory response (31). The hypothesis of donor-specific injury is supported by recent studies: Baciu and colleagues found that graft injury in DBD donors was characterized by inflammation, whereas in DCD graft injury was characterized by cell death, apoptosis and necrosis (31); Duong and colleagues observed that NK cells and macrophages were higher in EVLP perfusate from DBD donor lungs, whereas memory T cells were enriched in EVLP perfusate from DCD donor lungs (32).

In contrast to the cold ischemic injury of conventional DBD, the underlying pathophysiological mechanisms of warm ischemic kidney injury encountered during DCD procurement may be related to anoxia, calcium overload, mitochondrial dysfunction, oxidative & nitrosative stress, immune response, and ultimately lead to cell death and graft dysfunction (33). Moreover, hypoxia-inducible factors (HIFs) are crucial transcription factors for adaptive hypoxic responses, orchestrating the transcription of numerous genes involved in angiogenesis, erythropoiesis, glycolytic metabolism, and inflammation (34). Stabilization of HIF has been linked to benefits in ischaemia–reperfusion injury (heart and liver), acute kidney injury, acute respiratory distress syndrome (ARDS), inflammatory bowel disease, infections, haemorrhagic shock, and renal anaemia. Hypoxia-inducible factor 1α (HIF1α)-dependent induction of the glycolytic system protects during ischaemia and reperfusion by promoting the metabolic survival of myocytes or through HIF1*α* interaction with circadian rhythm proteins. Notably, pharmacologically enhanced HIF stabilization by HIF-prolyl hydroxylase domain inhibitors has shown benefits in ischaemia- reperfusion injury (35, 36). Wang et al. identified the immune cells that accumulate in the liver within hours after transplantation in humans. They found that eosinophils play a hepatoprotective role and support healthy liver function following IR injury by producing interleukin-13 (IL-13) in response to IL-33 signaling. Promoting eosinophil recruitment to the liver could be an effective strategy to protect against hepatic IR injury (37). Additionally, acetaminophen treatment stabilizes hypoxia-inducible factor-2α (HIF-2α) in hepatic macrophages, reprogramming them to produce the hepatoprotective cytokine IL-6, which helps mitigate acetaminophen-induced liver injury (38).

Indeed, there is an urgent need for donor-specific injury optimized procurement, organ storage, organ perfusion, organ transport and evaluation strategies in solid organ transplantation. As current clinical practice optimizes donor procurement, assessment, and storage of conventional DBD, the donor-specific injury caused by warm ischemia and prolonged cold storage of DCD graft before organ transplantation must also be considered.

## 3 Conclusion

**Figure 1:**
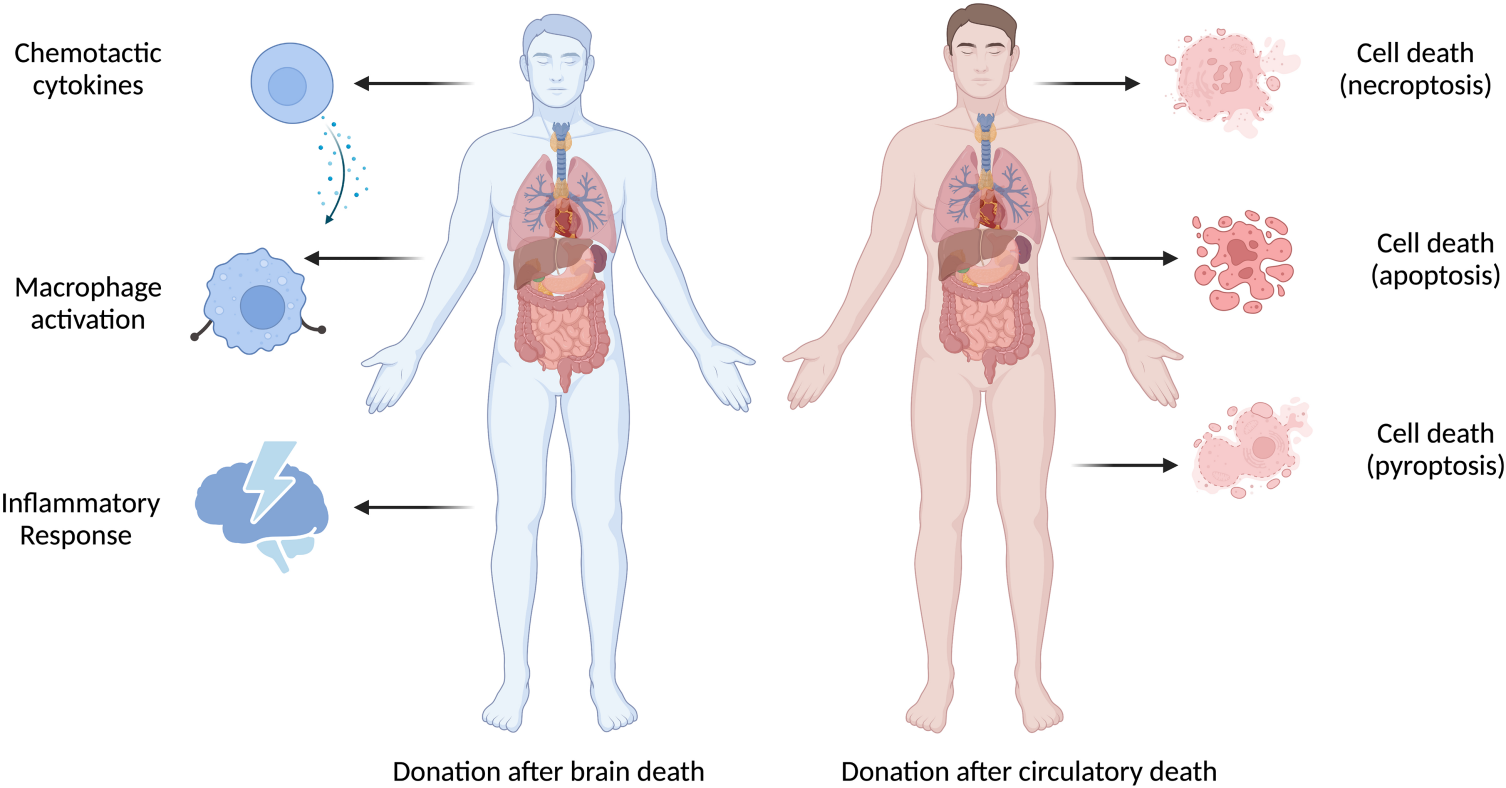
There are important differences between DBD and DCD donor-specific graft injury. Donor-specific graft injury is influenced by both the donor type and ischemia time, which is unique to each donor organ and causes various types of pathophysiologic consequences.

Taken together, these findings suggest that different types of donors may have different patterns of graft dysfunction and recovery, possibly requiring different therapies. *Ex vivo* organ perfusion is a promising technique to allow the functional evaluation, therapeutic repair of injured organs, cellular and molecular assessment of marginal donor organs prior to transplantation. While this technique has been widely adopted in various European and North American countries, in-depth studies and clinical trials are still necessary to evaluate the potential of this evolving field in improving graft function and transplant outcomes. Future clinical studies should consider anti-inflammatory therapies to improve graft quality in DBD allografts and targeted therapies for cellular death to allow successful rehabilitation and transplantation of DCD allografts (Figure 1). HIF could be protective during ischemia and reperfusion injury, as targeting cardiac HIF1α prevents the cardioprotective effects of ischaemic preconditioning. DCD donors will continue to be an important source of life-saving organ transplants as the demand for organ transplants continues to grow worldwide. To better utilize marginal donor organs, it is critical to fully understand the underlying mechanisms of donor-specific graft injury and how to improve graft function. Here, we recommend that future studies investigate and report donor type-specific graft injury at the molecular level, to provide insights into the underlying pathophysiology, associated biomarkers, and actionable drug targets.
